# A cell culture model using rat coronary artery adventitial fibroblasts to measure collagen production

**DOI:** 10.1186/1471-2261-7-13

**Published:** 2007-05-08

**Authors:** Cathleen Jenkins, Amy Milsted, Kathleen Doane, Gary Meszaros, Jonathan Toot, Daniel Ely

**Affiliations:** 1 Kent State University, Kent, Ohio, USA; 2 The University of Akron, Akron, Ohio, USA; 3 Northeastern Ohio Universities College of Medicine, Rootstown, Ohio, USA

## Abstract

**Background:**

We have developed a rat cell model for studying collagen type I production in coronary artery adventitial fibroblasts. Increased deposition of adventitial collagen type I leads to stiffening of the blood vessel, increased blood pressure, arteriosclerosis and coronary heart disease. Although the source and mechanism of collagen deposition is yet unknown, the adventitia appears to play a significant role. To demonstrate the application of our cell model, cultured adventitial fibroblasts were treated with sex hormones and the effect on collagen production measured.

**Methods:**

Hearts (10–12 weeks) were harvested and the left anterior descending coronary artery (LAD) was isolated and removed. Tissue explants were cultured and cells (passages 2–4) were confirmed as fibroblasts using immunohistochemistry. Optimal conditions were determined for cell tissue harvest, timing, proliferation and culture conditions. Fibroblasts were exposed to 10^-7 ^M testosterone or 10^-7 ^M estrogen for 24 hours and either immunostained for collagen type I or subjected to ELISA.

**Results:**

Results showed increased collagen staining in fibroblasts treated with testosterone compared to control and decreased staining with estrogen. ELISA results showed that testosterone increased collagen I by 20% whereas estrogen decreased collagen I by 15%.

**Conclusion:**

Data demonstrates the usefulness of our cell model in studying the specific role of the adventitia apart from other blood vessel tissue in rat coronary arteries. Results suggest opposite effects of testosterone and estrogen on collagen synthesis in the rat coronary artery adventitial fibroblasts.

## Background

Cardiovascular disease is the number one cause of death in the United States. Diseases such as hypertension and coronary artery disease develop through multiple mechanisms; of significance are the structural changes that take place within blood vessels. *In vivo *data has shown that coronary artery fibrosis can result as a consequence of high blood pressure (BP). In addition to elevated BP, testosterone has been shown to increase the amount of collagen measured around coronary blood vessels contributing to fibrosis. For example, Seachrist found that testosterone increased coronary adventitial collagen in male hypertensive rats independent of the renin-angiotensin system [1]. Studies have provided further evidence for the role of testosterone in coronary artery fibrosis in male borderline hypertensive rats. Sympathectomy reduced systolic BP, but more interestingly reduced plasma testosterone levels correlated with decreased coronary artery collagen deposition [2].

Historically, the adventitia has not been considered to be involved in the disease process associated with the blood vessel wall. In blood vessels, pathological changes have typically been attributed to detrimental alterations in endothelial function or the vascular smooth muscle (VSM) layer, whereas the role of the adventitia has been relegated to that of structural support. However, recent clinical and experimental research in coronary artery disease demonstrates that the adventitia does play an active role in the dynamic vascular remodeling in response to injury [3,4]. The adventitia participates in the response to endoluminal vascular injury in several animal models [5,6]. Female Sprague-Dawley rats subjected to carotid artery balloon injury showed migration of adventitial fibroblasts to the neointima [5]; and in the calf model, hypoxia induced a dramatic expansion of the pulmonary artery vaso vasorum via activation of fibroblasts [7]. Remodeling of the adventitial layer in canine coronary artery involves activation and proliferation of adventitial fibroblasts accompanied by expression of growth factors [7].

One structural change as a result of hypertension in both arteries and the heart is an increase in the amount of collagen. Collagen is synthesized by VSM cells, fibroblasts and myofibroblasts. Fibroblasts are usually credited for the majority of collagen in vascular tissue [8] and maintenance of the extracellular matrix, however, myofibroblasts are known to produce substantial amounts of collagen as well [9]. Fibroblasts can differentiate into myofibroblasts under a variety of stimuli and pathological conditions and alter collagen production [10]. For example, the cytokineTGF-β can induce the differentiation of fibroblasts to myofibroblasts and increase collagen production [11].

Collagen normally functions to impart structural integrity to the blood vessel wall, but in disproportionate amounts, it increases stiffness and blood vessel resistance, ultimately resulting in increased BP in hypertension. Besides collagen production, fibroblasts can function in a variety of ways. They are capable of migration to the injury site, pronounced proliferation, synthesis of other extracellular matrix proteins and vascular remodeling in response to injury or mechanical stress [12-14]. Besides structural functions, fibroblasts have an additional role through the release of cytokines or other regulatory molecules [15,16].

One limitation of *in vivo *studies is the inability to determine the role of the fibroblasts independent of the endothelium and VSM layers. Cell culture circumvents this problem and can be used to determine the role of fibroblasts in the absence of other cell types. Most cell culture models study collagen and fibrosis of the myocardium and aorta; however, the influence of coronary artery fibroblasts has not been adequately addressed, particularly fibroblasts from the spontaneously hypertensive rat (SHR), a well studied model of essential hypertension.

Thus the goal of this study was to culture adventitial fibroblasts from SHR coronary arteries to provide a tool for studying the effect of sex hormones on collagen as it relates to hypertension and vascular remodeling.

## Methods

### Animals

SHR were housed in polyethylene cages (45 × 25 × 20 cm) with stainless steel tops using heat treated wood chip bedding (P.J. Murphy Forest Products, Montville, NJ, USA). Rat chow (Teklad Rodent diet, Madison, WI, USA) and water were provided *ad libitum*. Animals were kept in constant conditions (25–27°C, 40–50% humidity, and a 12 hour light (0600–1800 h)/dark cycle (1800–0600 h). All procedures were in accordance with the National Institutes of Health guidelines on humane treatment of animals and approved by the University of Akron Institutional Animal Care and Use Committee.

### Tissue Harvest

**Figure 1:**
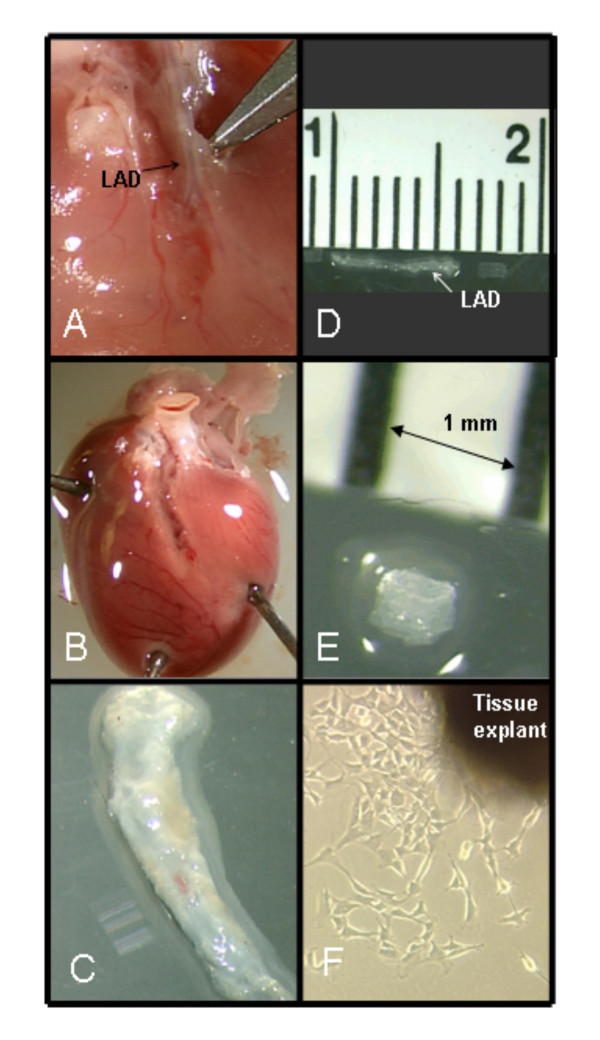
Removal of left anterior descending coronary artery (LAD) from SHR male rat. A) intact LAD, B) heart with LAD removed, C) isolated LAD, D) LAD length, E) LAD diameter, and F) fibroblasts migrating from LAD explant.

Adult SHR rats (250 g) (10–12 weeks) were anesthetized with 0.5 cc sodium pentothal (50 mg/ml, i.p.; Fort Dodge Animal Health, Fort Dodge, IA, USA), prepared for surgery, and the heart and thoracic aorta were removed and placed in cold, sterile buffer, either Kreb's Henseleit (Kreb's), Hepes buffered saline (HBS) or phosphate buffered saline (PBS). Hearts were pinned on sterile paraffin in petri dishes and sterile technique observed. The left anterior descending coronary artery (LAD) was isolated and removed from the heart (Figures 1A, 1B), maintained in buffer and dissected free of any extra tissue (Olympus SZX-12; Center Valley, PA, USA) (Figure 1C). The average size of the LAD was 6–10 mm in length and 0.5 mm in diameter (Figure 1D, 1E). Each LAD was sectioned into 3 mm pieces and placed in one standard 35 mm tissue culture dish. Due to anatomical differences between animals and the amount of branching of the left coronary artery, additional segments of blood vessels were collected from these coronary branches when possible.

For blood vessel dissection, early experiments attempted to process the LAD much like other blood vessels (aorta) [17,18], using collagenase and elastase digestion to facilitate the separation of the blood vessel layers. However the rat LAD is significantly smaller and has a reduced medial layer compared to the aorta, and when we attempted enzymatic separation of the layers we found it extremely rigorous and the tissue too fragile to yield viable cells. Consequently, enzyme digestion was not used to harvest fibroblasts from the rat coronary artery and intact tissue explants were plated. Explants were allowed to adhere to the culture dish in the absence of culture medium. After fibroblasts began to migrate, tissue explants were removed to minimize the introduction of contaminating cell types from the media or endothelial layers (Figure 1F). The time elapsed between removal of the heart and plating of the LAD explant was approximately 20–30 minutes.

In addition to LAD fibroblasts, fibroblasts were also cultured from the thoracic aorta and ventricle for comparative purposes. The thoracic aorta was cleaned of any additional tissue and flushed with buffer to remove any remaining blood. The vessel was then cut longitudinally, the endothelium gently scraped to remove any endothelial cells, cut into 4 × 6 mm sections and plated adventitial side down. Fibroblasts migrated from aortic tissue explants within 5–7 days at which time the explant was removed.

Ventricular fibroblasts were harvested from the heart using a modification of published methods [19]. Briefly, after LAD removal, the heart was placed in ADS buffer (116 mM NaCl, 20 mM Hepes, 1.0 mM NaH_2_PO_4_, 5 mM KCl, 0.8 mM Mg_2_SO_4_, 5.5 mM glucose, pH 7.4) and the large vessels and atria were removed. The ventricles were then minced, placed in a Wheaton CEL-STIR flask and digested with trypsin (0.6 mg/ml) and collagenase (100 U/ml) (both from Worthington Biochemicals, Newark, NJ, USA) in ADS buffer. Fetal bovine serum was added to stop digestion, the cell suspension centrifuged and the remaining pellet resuspended in 5 ml cell culture medium and plated to a 60 mm plate. Cardiac fibroblasts were allowed to attach to culture dishes for 45 minutes before medium was removed and transferred to a second 60 mm tissue culture plate. Fresh medium was added to the original plate and the same procedure was followed for the second 60 mm plate. Non-adherent cells were washed away 24 hours later.

### Cell Culture

Combinations of tissue culture medium were tested: Ham's F12K and Dulbecco's Modified Eagles Medium (DMEM; Invitrogen, Carlsbad, CA, USA) separately and a 50/50 combination of the two. All medium was supplemented with antibiotic/antimycotic (2 U/ml penicillin, 2 μg/ml streptomycin and 5 ng/ml amphotericin B; Sigma, St. Louis, MO, USA). Fetal bovine serum (FBS) concentration was incrementally decreased (20 -15 -10 percent) with each passage. Cell preparations were kept at 37°C in a humidified atmosphere of 95% air/5% CO_2_. Cells from early passages (P_2 _to P_4_) were cultured on microscope slides at a subconfluent density of 5 × 10^4^/well (11.5 mm diameter; Erie Scientific, Portsmouth, NH, USA), incubated overnight to allow for attachment, and subjected to immunohistochemistry (IHC) for cell verification or subjected to drug treatments.

### Cell Verification

Verification and viability of the fibroblastic cell line was determined by IHC staining. Cells were incubated 1 hr at 37°C with one of the following antibodies: mouse anti-vimentin directly conjugated with Cy 3 (1:400), mouse anti-α smooth muscle actin (α-SMA) antibody directly conjugated with FITC (1:1000), or primary mouse anti-desmin antibody (1: 200) with secondary horse anti-mouse IgG FITC labeled antibody (1:100)(all were from Sigma, St. Louis, MO, USA). VSM cells harvested from male SHR aorta were used as positive controls. Stained cells were viewed with an Olympus B-Max 60 microscope equipped with epiflourescence (Olympus, Center Valley, PA, USA) and pictures acquired digitally with a Retiga cooled CCD camera (Q Imaging, Burnaby, BC, Canada).

### Drug Treatment

To demonstrate that this model for culturing LAD fibroblasts has a direct application in vascular studies, fibroblasts were treated with sex hormones to induce changes in collagen type I production. For IHC, fibroblasts were plated on slides as described above and incubated in serum free, phenol red free media for 24 h prior to treatment. Cells were treated for an additional 24 h with either 10^-7^M testosterone or 10^-7^M estrogen (both from Sigma, St. Louis, MO, USA) and subsequently immunostained for collagen type I with rabbit anti-rat collagen type I polyclonal antibody (1:40; Chemicon, Temecula, CA, USA) and fluorescein labeled secondary antibody (1:100; Vector Laboratories, Burlingame, CA, USA). For quantitative collagen measurements, fibroblasts were plated at a density of 6 × 10^3 ^in a 96 well tissue culture treated microtitre plates (Becton -Dickinson). After 24 h incubation, basal culture medium was replaced with serum free, phenol red free medium for an additional 24 hours. Fresh serum free phenol red free medium with either 10^-7^M testosterone 10^-7^M estrogen was then added for 24 h of drug treatment.

### Quantification of Collagen Type I

ELISA techniques were used to measure collagen type I. Briefly, cells were fixed in 3.7 % formaldehyde for 10 minutes at room temperature (R.T.), washed and permeabilized with Triton X- 100 (0.3% in PBS) for 10 minutes at R.T. and washed again. All washes were conducted with a 0.1% Tween 20 in PBS. A blocking agent (2.5% BSA in PBS/Tween 20) was added to each well and incubated for 1 hour at R.T. or overnight at 4°C and then washed before adding the primary antibody. Cells were then incubated for 1 hour at 37°C with 50 μl/well of rabbit anti-rat collagen type I polyclonal antibody (1:500; Chemicon, Temecula, CA, USA), washed and then incubated 1 hour at 37°C with a goat anti- rabbit IgG secondary antibody conjugated with horseradish peroxidase. Plates were washed and 200 μl of substrate solution made with Sigma FAST OPD (*o*-phenylenediamine dihydrochloride) tablets dissolved in 20 ml of distilled water. Final concentrations are 0.4 mg/ml OPD, 0.4 mg/ml urea hydrogen peroxide, and 0.05 M phosphate-citrate, pH 5.0. The enzyme reaction was stopped after 30 min incubation at room temperature (in the dark) by the addition of 50 μl of 2 M H_2_SO_4 _to each well. Plates were then read at 450 nm (SoftMax Pro software) on a SpectraMax Plus multiwell plate reader (Molecular Devices).

## Results

### Tissue Harvest

During dissection we found that minimal manipulation of the LAD ensured that cells were more likely to migrate from the tissue explant. Initially, different buffers (Kreb's, HBS, or PBS) were tested to determine if the different buffering capacity (bicarbonate vs. Hepes) had any effect on tissue viability. Explants processed in PBS (~40–50% success rate) did not produce as many cells as those maintained in Kreb's or HBS (~80–90% success rate) (Table 1). However, there were no observable differences between Kreb's and HBS buffers used during dissection which changed any experimental results. If explants did not adhere to the culture dish, then no cells were obtained from that explant.

### Timing and Age of Animal

We established the optimal time from the removal of the heart to the time when the LAD was plated for cell culture. In preliminary experiments the time elapsed was as great as 2 hours. The longer the time lapse between the removal of the heart and the plating of the vessel, the less successful the culture. The minimum amount of time from vessel removal to culture medium was approximately 25 minutes and proved to yield the greatest number of cells, indicating the importance of speed at this stage (Table 1).

In younger animals (10–12 weeks of age) 80–90% of the explants survived. Cultures from older animals (18–30 weeks of age) did not thrive and typically only 25–30% of the explants produced cells and in the oldest animals (30+ weeks of age) no cells survived (Table 1).

### Proliferation

The proliferation rate was different when comparing fibroblasts from LAD, aorta and ventricle. In early experiments explants from LAD and aortic fibroblast were both started (P_0_) in 35 mm dishes for comparison purposes. The average number of fibroblasts from passage one (P_1_), LAD had 4 × 10^5 ^cells/11.78 cm^2 ^in one 35 mm culture dish in contrast to the aorta which had a total of two 35 mm dishes each with an average of 2 × 10^6 ^cells/11.78 cm^2^. Those same cultures were maintained to passage 3 (P_3_) and the average cell number was 6 × 10^5^/11.78 cm^2 ^still in one 35 mm dish, whereas the number of P_3 _aortic fibroblasts had increased to two 60 mm dishes each with 3 × 10^6^/21.29 cm^2 ^cells.

To demonstrate the difference in the total number of fibroblasts and rates of proliferation, cells were counted in later experiments where we attempted to harvest the maximal amount of fibroblasts from each tissue. LAD explants yielded the same number of cells as mentioned above but since the amount of aortic tissue available was greater so is the potential for more fibroblasts. In fact, if 10 aortic explants were started (P_0_) in a 100 mm dish there was 1.4× the number of P_1 _fibroblasts compared to LAD and 3× the number of P_3 _fibroblasts (Table 2). Comparing the number of fibroblasts from both ventricles to the LAD, there were 9× more cells at P_1 _and 364× more cells at P_3_. The change in the number of cells from P_1 _to P_3 _demonstrates how different the rate of proliferation was for each tissue. The number of cells increased 1.5× in LAD compared to 3.2× in aorta and 60.7× in ventricles (Table 2).

Once the initial explant was established, it took 5–7 days for fibroblasts to migrate out from the explant and to increase in numbers large enough to sustain themselves once the explant was removed (7–10 days) (P_0_). At that time cells were often clumped in dense patches, so they were trypsinized to evenly disperse them into another 35 mm dish (P_1_). After an additional 4–7 days, if cells were confluent they were passed in a 1:2 dilution (P_2_) to another 35 mm dish. However, if cells had not reached confluency (~85%) at this time, it was important to pass them without allowing additional time in the same culture dish. The vigor needed to remove P_1 _or P_2 _cells that had been plated more than 7 days resulted in increased cell death. Because of this, P_1 _cells at less than 85% confluency were passed at the 4–7 day point to 2–3 wells in a 12 well plate instead of a 35 mm dish. This provided a smaller growth area of 3.8 cm^2 ^compared to 11.78 cm^2 ^and ensured an optimum cell density. After another 4–6 days cells were passed (P_3_) if they were actively dividing. More consistent results were found when using early passage cells (P_2 _to P_3_) for drug treatments and the optimal age of the fibroblasts appears to be from 15–24 days after removal from the animal. Proliferation decreased dramatically at or beyond P_3 _and cell morphology was altered. For instance, the cells were more firmly attached to the substrate, had increased contrast compared to younger cells and were flatter with cytoplasmic extensions. The ratio of cytoplasmic area compared to nuclear area was much greater in older cells.

### Culture conditions

Composition of the culture medium was another important factor in cultivating fibroblasts. Coronary artery fibroblasts grew best in the 50/50 mixture of DMEM and Ham's F12K. All culture medium was supplemented with FBS and antibiotic/antimycotic solutions. The explant cultures supplemented with 20% FBS were more successfully established than those initiated with only 10% FBS, therefore 20% FBS was used in the medium for explants and P_0 _cells. As the cells were subcultured, the amount of FBS was reduced incrementally to 15% and 10%, respectively, prior to drug treatments. The reason for this procedure was that the immediate change from 20% FBS to serum free media was too severe and resulted in increased cell death.

Experiments were conducted when P_1 _fibroblasts were passed into either one 60 mm dish, multiple 12 wells or 35 mm dishes until we realized that cells in the larger dish stopped dividing. LAD explants initially started in 35 or 60 mm dishes and were only successful in the 35 mm plates.

### Cell Characterization

**Figure 2:**
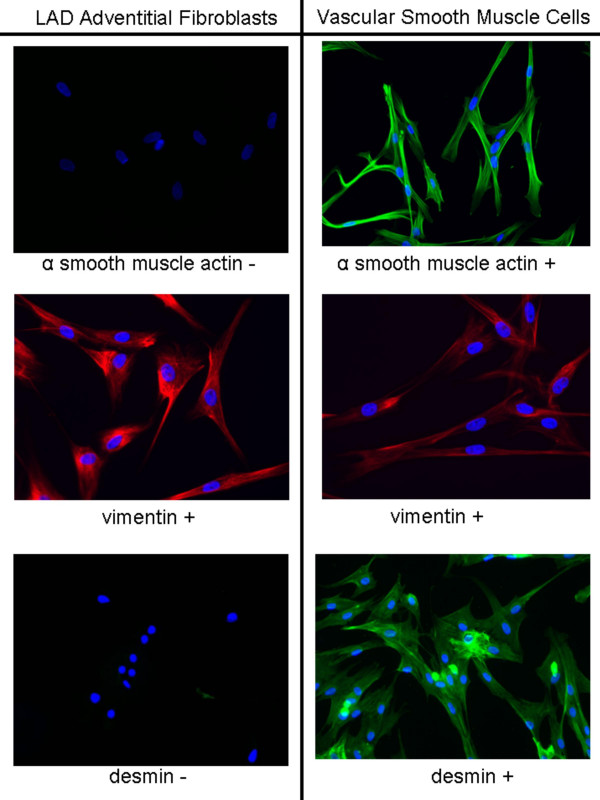
Cell Characterization. Cultured rat coronary artery adventitia fibroblasts (LAD) and aortic vascular smooth cells (VSM) (positive control) stained for desmin, α-smooth muscle actin (α-SMA) and vimentin. LAD stained negative for desmin and αSMA and positive for vimentin, a fibroblast marker. VSM stained positive for all 3 proteins. Desmin and α-SMA are specific VSM markers.

Since explants were from intact blood vessels, all tissue layers were present. Consequently, cell types cultured were verified using IHC to ensure the cells tested were indeed fibroblasts. Fibroblasts stained positive for vimentin, but not for α-SMA or desmin. VSM cells from male SHR aorta were used as positive controls and stained positive for all three cytoskeletal proteins: α-SMA, desmin and vimentin (Figure 2). The absence of endothelial cells was confirmed morphologically. It should be emphasized that the cells used in this study were from early passages. Cells that were maintained longer at P_3 _and into P_4 _or plated at lower densities showed positive staining for α-SMA indicting the presences of myofibroblasts (data not shown).

### Qualitative Measure of Collagen Type I

**Figure 3:**
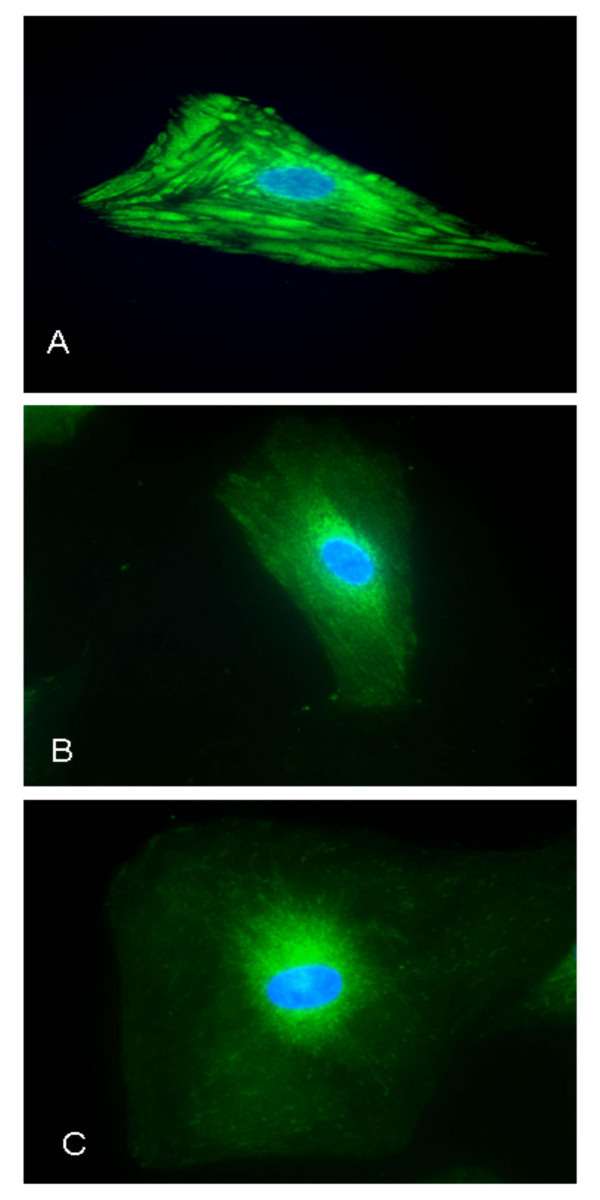
Cultured fibroblasts immunostained for collagen type I after 24 h exposure to 10^-7^M testosterone or 10^-7^M estrogen. A) Testosterone increased the amount of collagen present compared to control (C) as indicated by the increased number of secretory vesicles and the intensity of staining. B) Estrogen did not have any significant effect on collagen production.

LAD fibroblasts treated with 10^-7^M testosterone for 24 h showed an increase in the intensity and amount of collagen type I stained as well as more pronounced secretory vesicles in stimulated cells (Figure 3A). Cells treated with 10^-7^M estrogen for the same time period appeared similar to control (Figure 3B).

### Quantitative Measure of Collagen Type I

**Figure 4:**
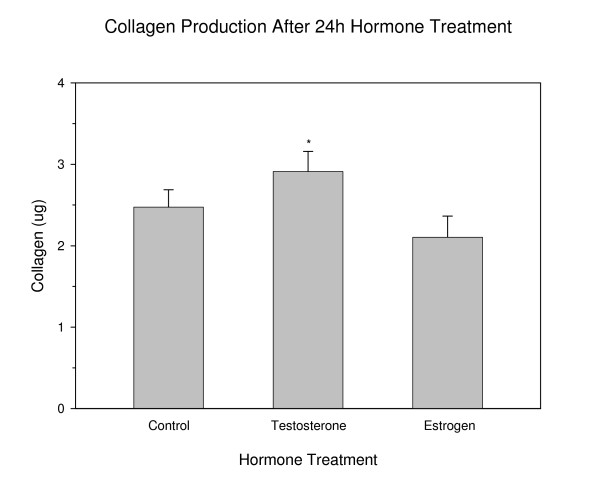
Collagen Type I (μg) measured by ELISA in LAD fibroblasts treated with 10^-7^M testosterone or 10^-7^M estrogen. Testosterone significantly increased the amount of collagen deposited (2.912 ± 0.247 μg) compared to control values (2.475 ± 0.211 μg) (* p < 0.05, 1 tailed t-test). Estrogen decreased the amount of collagen (2.103 ± 0.262 μg) compared to control (p = 0.058, 1 tailed t-test). Collagen was measured in wells with a 0.32 cm^2 ^growth area from a 96 well culture plate.

Cells treated with testosterone (N = 22 animals, 84 wells) had 2.912 ± 0.247 μg/well of collagen type I compared to control values 2.475 ± 0.211 μg/well (N = 26 animals, 132 wells) (Figure 4). This represents a 20% increase in the amount of collagen type I deposited by the fibroblasts in a 24 hr period (p = 0.04, 1- tailed t test).

However, cells treated with estrogen (N = 18 animals, 81 wells) showed the opposite effect compared to testosterone by decreasing the amount of collagen deposited by 15%. Estrogen treated cells produced 2.103 ± 0.262 μg/well compared to control of 2.475 ± 0.211 μg/well (p = 0.058, 1 tailed t test) (Figure 4).

## Discussion

The initial questions of this study asked whether it was feasible to culture rat coronary artery adventitial fibroblasts for experimental use, and can we demonstrate the utility of this technique for measuring changes in collagen as related to vascular remodeling and hypertension.

The most important outcome of this study is that we established and optimized a new culture system for LAD fibroblasts that provides a practical means for studying cells involved in blood vessel pathology. Our data illustrates the response of adventitial fibroblasts to sex hormones in the absence of other vascular cell types. Collagen type I production was 20% higher in testosterone treated cells compared to control fibroblasts. This significant increase in collagen *in vitro *mirrors the results we found through *in vivo *experiments conducted in our lab. Previously we have shown that testosterone has a role in the development of hypertension as well as the resulting pathology that can arise in coronary blood vessels. For instance, castration of hypertensive, borderline hypertensive and normotensive rat strains significantly reduced blood pressure (20–30 mmHg) and testosterone replacement in castrated males restored BP in all strains [20]. We have also looked at the effect of testosterone on coronary collagen deposition. Research has shown the effect of the renin-angiotensin system (RAS) is to increase collagen production in cardiac fibroblasts and when subjected to an angiotensin-converting enzyme (ACE) inhibitor collagen production is decreased [21,22]. We have had similar results using an ACE inhibitor which reduced coronary artery collagen [1]. In the same study, we found a significant positive correlation between collagen and plasma levels of testosterone. This finding indicates a separate and independent effect of testosterone over the RAS in collagen production.

In addition we have researched the role of the sympathetic nervous system (SNS) in hypertension and associated fibrosis. We found that sympathectomized rats show a decrease in plasma testosterone and the absence of the increase in collagen deposition typically seen around the coronary arteries [2]. Besides the histological effect, we also have demonstrated a link between norepinephrine and the pathology associated with high BP and the SNS. We used testosterone implants in both castrated normotensive and castrated hypertensive rats to show that there is a direct relationship between the levels of norepinephrine and testosterone [23]. To further substantiate the role of testosterone and BP, our studies of the androgen receptor have shown that rats with deficient androgen receptors do not exhibit the same BP changes as those animals with functional receptors [24]. The data when taken together demonstrate that testosterone has a role in the coronary pathology seen with hypertension.

Another interesting finding in this study was that LAD fibroblasts are affected in opposite ways by testosterone and estrogen. A 15% decrease in collagen was measured in fibroblasts treated with estrogen (Figure 4). This finding is consistent with the reported vasoprotective effects of estrogen [25,26] indicating that 17β-estradiol and progesterone may protect postmenopausal women against cardiovascular disease [27]. In cardiac fibroblasts, drugs that blocked the metabolism of estrogen intermediates blocked the inhibitory effects of hydroxyestradiol on cardiac fibroblast proliferation and collagen synthesis [28,29]. A possible explanation for the different effects of testosterone and estrogen may be that they are acting in a different way on the synthesis and degradation of collagen I. If the ratio between the two were altered the relative amount of collagen I would change.

Besides illustrating the usefulness of this culture system, another important outcome was determining the ideal conditions to culture and maintain LAD fibroblasts while maximizing cell yield. We found that the conditions and timing of tissue harvest, the composition of culture medium, culture dish size and the age of the donor animal were all key variables.

The conditions of the tissue harvest and degree of vessel manipulation proved to be critical. Three different buffers, PBS, HBS and Kreb's were all used to maintain the tissue while harvesting the coronary artery. Tissues initially maintained in PBS ultimately had fewer cells compared to those kept in the other buffers. The decreased number of cells from explants processed in PBS could be due to the differing composition of the buffers or that the PBS was used initially when the technique was being developed and other conditions had not been optimized. The fact that the minimal amount of time from vessel removal to culture media provided the greater number of cells is of no surprise. Less time in buffer and a nutrient deficient environment should minimize tissue damage and cell death.

Another important factor was age of the animal. Developmentally, many tissues consist of terminally differentiated cells that become quiescent after a fixed number of cell divisions. In addition to their cell division potential, many cells become metabolically inactive after a specific time, explaining why explants from younger animals (10–12 weeks) yielded more cells. Animals less than 10 weeks would most likely be viable in terms of age; however, the younger animal provides less tissue which becomes a limiting factor in the total number of cells yielded.

Not only is the total number of cells generated from LAD explants a limiting factor due to the amount of tissue available from one animal, but the rate at which the cells proliferate is a constraint as well. In comparison to aortic and ventricular fibroblasts, the growth rate of LAD fibroblasts appears to be the slowest, taking longer to reach confluency and have the fewest total number of cells. The overall number of cells was dramatically different and most likely due to the relative amount of tissue available and the different growth rates associated with fibroblasts of different origins. This finding is not surprising considering that studies have shown that fibroblasts exist in heterogeneous populations in which they have very specific phenotypes and functions based on the tissue of origin [30] and rat strain [31]. The decrease in cell division is consistent with the changes seen in aging fibroblasts from other sources such as human gingiva or foreskin [32,33], reflecting that most cells have a finite period of viability and are not immortal [34].

The morphological changes seen in later passage cells could be explained by the decrease in cell division and overall numbers of cells allowing the cells that were present to spread out more creating a greater cytoplasmic area to nuclear area ratio. The difficulty in removing these cells for subculture was most likely due to the increased deposition of extracellular matrix while in the culture dish for a longer period of time.

Cell density and culture dish size were important as well. Fibroblasts have been shown to be sensitive to cell density in culture [35]. In rabbit corneal fibroblasts, Masur showed that cell density was a critical factor in both fibroblast proliferation, as well as transformation of fibroblasts to myofibroblasts [35]. In cultures where cells were seeded at a less than optimal density, fibroblasts began to differentiate into myofibroblasts and the rate of cell division decreased. Our findings are consistent with this in that the size of the culture dishes used when passing these fibroblasts directly impacted cell density since there is initially a limited number of cells. In other studies we have found the presence of myofibroblasts in LAD fibroblast cultures that were plated at lower densities as well as in older cultures (late passage 3 and 4) (data not shown). Older cells or those plated at less than optimal density stained positively for α-SMA indicating that they had differentiated to myofibroblasts. However, the cells in this study were plated in 35 mm culture dishes and used while younger in early passages. Fibroblast cell type was confirmed by positive staining for desmin and negative staining for α-SMA. This demonstrated that the cultures in this study were predominantly fibroblasts and not cells that had transformed into myofibroblasts.

## Conclusion

In summary, we have demonstrated the following: 1) a procedure for successful isolation and culture of rat LAD fibroblasts; 2) an optimal timeline for rat fibroblast migration and proliferation; 3) an experimental design for hormone treatment of LAD fibroblast cell cultures; and 4) testosterone increased collagen type I and estrogen decreased collagen type I in LAD fibroblasts. We provide a useful technique for isolating coronary artery adventitial fibroblasts and have demonstrated the utility of this technique in experimentally determining their influence in blood vessel physiology and pathology.

## Competing interests

The authors declare that they have no competing interests.

## Authors' contributions

CJ conducted research and primary author of paper. AM provided cell culture expertise and participated in design of study and edited manuscript. KD provided immunohistochemistry and fibroblasts culture expertise and assisted in photography of cell. GM provided the expertise and equipment for isolating ventricular fibroblasts. JT assisted in data analysis and helped to edit draft of manuscript. DE is senior investigator for our lab and was the primary editor of manuscript.

## Pre-publication history

The pre-publication history for this paper can be accessed here:



## Figures and Tables

**Table 1:** Explant Success by Experimental Variables

	**Type of Buffer**		
	PBS	HBS	Kreb's
**Percent of successful explants**	40–50%	80–90%	80–90%

	**Time from Tissue Harvest to Culture**		

	< 1 hr	1–2 hrs	> 2 hrs
**Percent of successful explants**	80–90%	50–60%	0–5%

	**Age of Animal**		

	10–12 wks	18 wks	> 30 wks
**Percent of successful explants**	80–90%	25–30%	0%

**Table 2:** Cell Density and Fold Change by Tissue and Passage Number

	**Cells/cm**^**2**^		**Fold Change**		
	**Passage 1**	**Passage 3**	**P**_**1**_**-P**_**3**_	**P**_**1 **_**(compared to LAD)**	**P**_**3 **_**(compared to LAD)**

**LAD**	3.4 × 10^4^	5.0 × 10^4^	1.5 ×	---	---
**Aorta**	4.7 × 10^4^	15.2 × 10^4^	3.2 ×	1.4 ×	3.0 ×
**Ventricle**	3.0 × 10^5^	18.2 × 10^6^	60.7 ×	9.0 ×	364 ×
